# Stop adding insult to injury—identifying and managing risk factors for the progression of acute kidney injury in children

**DOI:** 10.1007/s00467-017-3598-3

**Published:** 2017-02-15

**Authors:** Wesley Hayes

**Affiliations:** 1 Great Ormond Street Hospital for Children, London, UK; 2 University College London, London, UK

**Keywords:** AKI, Children, Progression, CKD, Hypertension

## Abstract

Acute kidney injury (AKI) is common in children admitted to hospital. Whilst some recover normal kidney function following an acute kidney insult, a significant proportion experience long-term sequelae. The aim of this review is to summarize current understanding of the processes that can lead to sequelae following AKI. Kidney injury, repair, recovery and progression are described. Risk factors for progression are outlined, and potential strategies to stratify the risk of progression in children with AKI are discussed. Clinical management priorities to minimize sequelae are suggested. Looking ahead, novel therapeutic targets are discussed with the potential to accelerate adaptive repair and ameliorate the progression and sequelae of AKI in the future.

## Introduction

The incidence of acute kidney injury (AKI) in children is rising. One in three children worldwide experience AKI during an episode of hospital care [1]. Most children are exposed to AKI risk factors, such as medication with nephrotoxic potential, during a hospital stay [2, 3]. To date, initiatives have rightly prioritized improving the identification and initial management of AKI with global awareness-raising campaigns and national and international diagnosis and management guidelines [4–6]. The events that follow AKI have received less attention.

Traditionally, AKI in children had been understood to be an entirely reversible phenomenon. In recent years, this customary view has been challenged by emerging evidence that supports a strong link between AKI and chronic kidney disease (CKD) in adult patients [7]. In this review, current understanding of the events that follow acute kidney insults in children is outlined, namely renal injury, repair, potential recovery, or progression to long-term sequelae. These concepts are defined, and their underlying mechanisms outlined. Risk factors for progression are described, with discussion of clinical management priorities and potential future therapies aimed at minimizing long-term sequelae following AKI in children.

## Long-term sequelae of pediatric AKI

Children have the potential to recover ostensibly normal kidney function following an episode of AKI, however a growing body of evidence suggests that chronic sequelae may be under-recognized. Over one-third of 176 children treated for AKI at a U.S. tertiary center had reduced kidney function, or remained dialysis dependent, at the time of discharge from hospital [8]. In the 3- to 5-year follow-up of this cohort, 17 of 29 children had long-term effects, including hyperfiltration, reduced kidney function, hypertension or proteinuria [9]. In another follow-up study of 126 children treated in intensive care, 10% had CKD within 1–3 years following AKI [10]. A U.S. study of 63 pediatric heart transplant recipients with AKI showed that 5% of the patients had developed CKD at 12-month follow-up [11], and six of 37 children treated for AKI at a tertiary center in India had abnormal renal parameters at 10-year follow-up [12]. Infants who experience AKI in the neonatal period have an increased risk of abnormalities in kidney function in the long term [13, 14]. Taken together, these data demonstrate that recovery of normal kidney function following AKI in children is by no means guaranteed; pediatric AKI cannot be treated as an isolated event without due attention paid to the potential consequences.

Some children do recover their baseline kidney function following AKI, however understanding functional renal recovery in children is challenging for a number of reasons. Firstly, pediatric follow-up studies to date have not applied a standard definition of renal recovery, with definitions varying from dialysis independence during a hospital stay through to normalization of plasma creatinine, blood pressure and proteinuria. Secondly, the natural history of increasing glomerular filtration rate (GFR) in the first year of life from 15 to 90 ml/min/1.73 m^2^ presents challenges in defining baseline kidney function for infants. Thirdly, children have substantial renal reserve, so significant kidney damage resulting from an AKI episode can be compensated by hyperfiltration and may therefore not be reflected in the plasma creatinine level until adolescence or adulthood [15]. These challenges highlight the need to look beyond an operationalized definition of renal recovery based on functional measures, such as creatinine, in order to identify children who are at greatest risk of long-term sequelae following AKI. The identification of such children requires a deeper understanding of the sequence of events that follow an acute kidney insult, namely injury, repair and potential progression.

## The events that follow an acute kidney insult

**Fig. 1:**
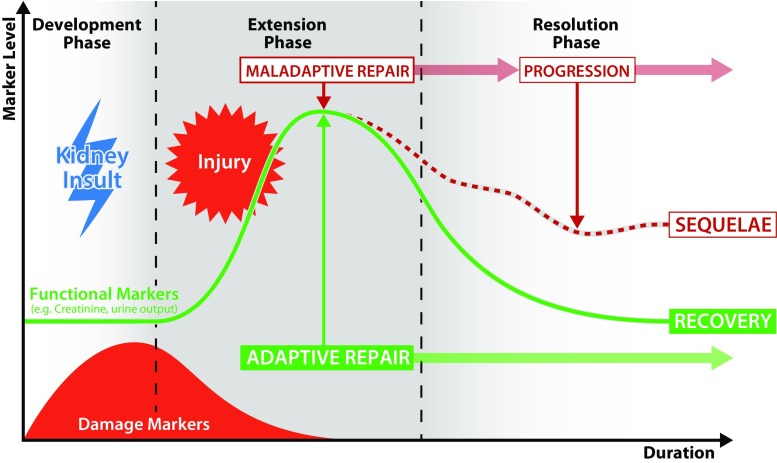
The sequence of events following an acute kidney insult

Until recently, progress in characterizing the events that follow an acute kidney insult was hampered by the lack of a consensus definition of progression and its associated events. Following recognition of this issue, a working group of the 13th Acute Dialysis Quality Initiative (ADQI) Conference sought to clarify these concepts. This group outlined a paradigm for understanding the clinical course of AKI and proposed definitions for terms related to events mediating resolution or progression [16]. They conceptualized the events that follow an acute kidney insult in three phases, as illustrated in Fig. 1. The development phase represents the immediate effects of the initial insult, which may be subclinical. The extension phase then ensues, in which both injury from the kidney insult and repair mechanisms compete. The resolution phase represents the net outcome of damage and repair. The duration of each phase can vary considerably depending on the nature of both the kidney insult and repair processes.

Concepts relating to progression following AKI are best understood in the context of the sequence of events described above. In the development phase, a kidney insult leads to injury. In the extension phase, repair processes are initiated in response to injury. Adaptive repair is a coordinated process, resulting in the resolution of renal structure without long-term sequelae. In contrast, maladaptive repair results in durable reduction in kidney function usually associated with a change in renal structure. The extension phase represents the net result of renal injury and repair processes. Renal recovery is a consequence of adaptive repair that leads to durable improvement in kidney function or structure. Conversely, progression is defined as a durable change in kidney structure or function detected by biomarkers, imaging studies or histopathology [16].

Recent progress has been made in operationalizing a definition of renal recovery; the Kidney Disease: Improving Global Outcomes (KDIGO) AKI work group developed the entity “acute kidney disease” to characterize partial functional recovery following AKI [5]. This was defined as a GFR of <60 ml/min/1.73 m^2^ or evidence of structural kidney damage for less than 3 months. This concept bridges the gap between the “Loss” and “End Stage” categories of the pediatric RIFLE criteria [17]. In clinical practice, recovery is often thought of as a return to baseline plasma creatinine. As discussed above, functional renal recovery should be interpreted with caution in children, as a return to baseline creatinine may mask underlying durable kidney damage.

## Pathophysiology of progression following AKI

There is a growing understanding of the pathophysiologic mechanisms that underlie progression following AKI. Acute kidney insults disrupt tubules, capillaries and glomeruli, with the proximal tubule being particularly vulnerable [18]. Repair processes are then initiated in response to the insult [19, 20]. Sustained recovery can be attributed to adaptive repair processes resulting from a well-balanced response between inflammatory and anti-inflammatory factors [21]. In many situations, this fine balance is not achieved, resulting in maladaptive repair, which predisposes to the development of interstitial fibrosis. Adaptive and maladaptive repair can be focally variable; tubular damage can resolve completely in some areas, whereas other areas can be less resilient to insults, leading to tubular atrophy. Maladaptive repair and interstitial fibrosis reduce the kidneys’ reserve available to buffer further kidney insults. A negative spiral can then ensue, in which fibrotic damage further increases the risk of subsequent progression and more extensive chronic damage. The finely balanced response to the initial kidney insult, together with the nature and duration of this insult itself, are critical in determining the long-term outcome following AKI [7, 16, 22].

**Fig. 2:**
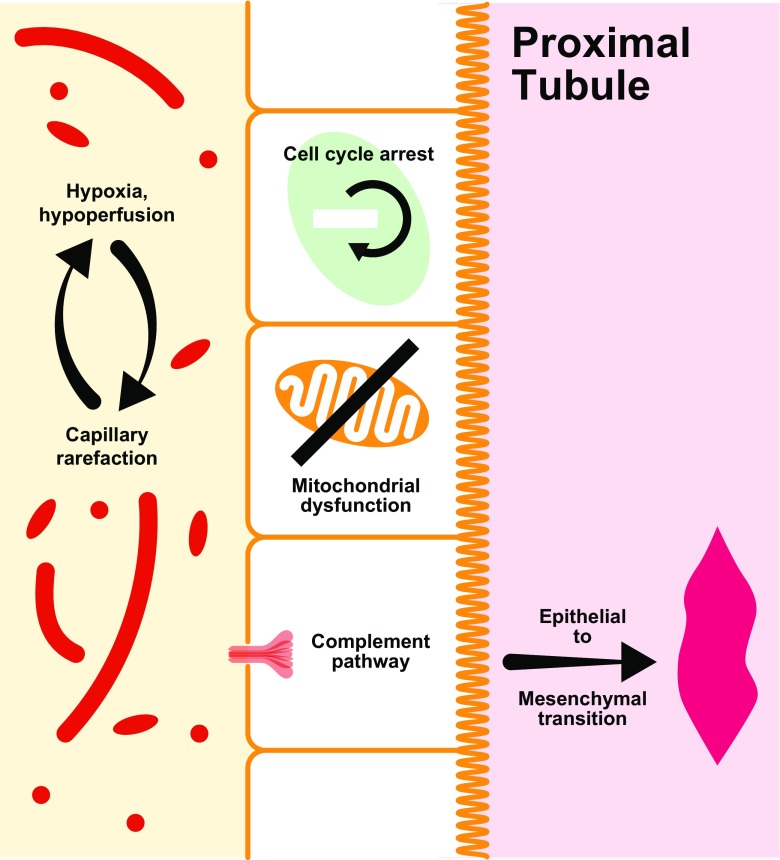
Pathophysiologic mechanisms of progression following acute kidney injury

Animal models of AKI have led to the identification of a number of mechanistic pathways that contribute to progression following an acute kidney insult. These include oxidative stress, the DNA damage response pathway, epigenetic changes, mitochondrial dysfunction and the complement pathway, as illustrated in Fig. 2.

Oxidative stress plays a key role in both the development of kidney injury following an acute insult and maladaptive repair and progression following the injury itself [23, 24]. Notwithstanding the contribution of oxidative stress to injury and progression, there is some evidence that this pathway might be beneficial in ameliorating the effects of acute insults in specific circumstances. Ischemic pre-conditioning prior to a kidney insult has been found to reduce the development of AKI [25]. The likely mediators of this protective effect are cellular defense mechanisms, such as hypoxia-inducible factor (HIF) and nuclear factor erythroid 2-related factor 2 (Nrf2), which are activated by the pre-conditioning event [26]. Although potentially beneficial in ameliorating acute injury, pre-conditioning responses can be chronically deleterious [27]. Injury can lead to a reduction in capillary density that can exacerbate renal hypoxia and thereby contribute to progression [28, 29]. The durable effects of ischemic pre-conditioning in AKI require further careful evaluation.

The DNA damage response is a network of signaling pathways that can trigger cell cycle arrest, or cell death, in response to injury. DNA damage response pathways are activated by AKI [30]. Aberrant cell cycle arrest in proximal tubular cells has been implicated in maladaptive repair and the development of fibrosis [31]. Kidney injury can also influence the phenotypic transition of various cells that can lead to progressive damage. Transition of endothelial cells to a mesenchymal phenotype can impair proliferation and exacerbate oxidative stress [32]. Durable structural changes and progression can result from the transition of pericytes to myofibroblasts and from epithelial–mesenchymal transition in renal obstruction [33, 34].

Emerging evidence suggests a role for complement pathways in progression following an acute kidney insult. Knock-out of cell surface complement regulatory proteins from proximal tubular epithelial cells increases susceptibility to progressive injury in ischemia–reperfusion models of AKI [35, 36]. Complement pathway inhibition ameliorates kidney injury in this model. Further work is needed to clarify the exact nature of complement interactions in AKI progression.

It is anticipated that growing knowledge of the mechanisms that underlie progression following AKI will ultimately lead to therapies that may ameliorate long-term sequelae, as discussed further in the section “Future therapies which may ameliorate progression following AKI”.

## Identifying children who are at risk of progression following AKI

The identification of children who are most at risk of progression following AKI is necessary in order to target interventions to minimize progression. Risk factors for long-term sequelae following AKI and techniques to stratify the risk of progression in children are outlined in this section.

The severity of a kidney insult is a well-established risk factor for progression following AKI. Insults that are severe and/or prolonged can disrupt the balance of inflammatory and anti-inflammatory factors required to facilitate adaptive repair processes, and thus compromise renal recovery [37, 38]. Multiple acute kidney insults exert a cumulative impact that can hamper adaptive repair and lead to sequelae. Laboratory and clinical observations support this concept. In animal models, the frequency of proximal tubule injury affects renal prognosis [38]. In adult patients with diabetes mellitus, repeated episodes of AKI are associated with end-stage renal disease [39]. Multiple episodes of myohemoglobinuric AKI are closely associated with long-term kidney fibrosis and CKD [27]. The cumulative impact of multiple kidney insults can compromise adaptive repair processes with resultant progression.

Pre-existing CKD confers an increased risk of progression following AKI. Animal studies have demonstrated a propensity for maladaptive repair in the context of reduced renal mass [40]. Clinical studies in both adults and children evidence reduced capacity for functional renal recovery following AKI in patients with pre-existing CKD [7, 22, 41, 42]. This phenomenon represents a high-risk to children with CKD given their lifetime exposure to acute kidney insults.

Many of the risk factors for progression outlined above are challenging to quantify. Strategies with greater discriminative power to identify children at risk of progression are needed. Large data techniques in which electronic health information, such as laboratory values, vital signs and patient characteristics, are used to build prediction models for events have been successfully used to stratify adult patients at risk of developing AKI [43]. Further work is needed to evaluate the application of these techniques to determine progression risk following AKI in both adults and children.

The use of urinary biomarkers to inform the risk of progression following AKI has shown some potential. In adult patients, three biomarkers have been found to predict progression of AKI among patients with acute cardiorenal syndrome, namely urinary angiotensinogen, urinary neutrophil gelatinase-associated lipocalin and urinary interleukin-18 [44]. Whilst studies in neonates and children with AKI have identified biomarkers with some discriminative value for the early identification of AKI [45–49], their use in risk stratification for progression has not been evaluated to date.

Imaging applications have shown promise in identifying features that may predict progression following AKI. Two magnetic resonance imaging (MRI) techniques, namely diffusion-weighted-MRI and blood oxygen level-dependent-MRI, have been used to evaluate tubulointerstitial alterations and parenchymal hypoxia in patients with both AKI and CKD with some success [50]. Ultrasound and MRI elastography can provide hemodynamic and structural information which may be predictive of progression [51, 52]. Further evaluation of these non-invasive techniques may enhance assessment of progression risk in the future.

There is a clinical need to develop reliable strategies to stratify the risk of progression following AKI in the pediatric population in order to tailor appropriate therapy and follow up with the aim of minimizing long-term sequelae.

## Clinical strategies to minimize progression following AKI

Strategies to minimize progression following AKI in children have not been systematically studied to date. Interventions should target removal of kidney insults and optimization of conditions that facilitate adaptive repair. Clinical priorities include optimization of kidney perfusion, removal of iatrogenic insults, attention to urinary drainage and correction of acid–base and electrolyte disturbances.

### Optimizing kidney perfusion

Several factors underlie the kidneys’ vulnerability to malperfusion after an acute insult. Autoregulation of renal blood flow can be disrupted, compounded by falling oxygen tension in the renal medulla which is already relatively hypoxic under normal physiologic conditions. Repair processes are particularly vulnerable to hypoxia in the context of tubulointerstitial edema, vasoconstriction, endothelial injury and capillary compression in AKI [37]. Impaired production of vasoactive factors by damaged tubules, such as vascular endothelial growth factor (VEGF), further compromise parenchymal perfusion [53]. Malperfusion hinders metabolically demanding adaptive repair processes.

Clinical observations in adult critical care patients support the key role of optimal renal perfusion in promoting adaptive repair following AKI. Both higher systemic oxygen delivery and higher mean arterial blood pressure are independently associated with a lower risk of sequelae following AKI [54]. However, the optimization of kidney perfusion can be clinically challenging in the context of generalized fluid overload in children. Key principles include the maintenance of adequate systemic blood pressure, with careful attention to the assessment and correction of intravascular volume depletion. Excessive or over-rapid diuresis or ultrafiltation should be avoided. Inotropic support can be indicated, but there is no evidence to support the use of “renal dose” dopamine in AKI [55].

Novel therapies to reduce the impact of renal hypoperfusion on progression following AKI are on the horizon. These will be discussed further in the section “Future therapies which may ameliorate progression following AKI”. 

### Minimizing iatrogenic insults

Persistent or repeated kidney insults hamper adaptive repair. In children with AKI, urgent evaluation of all factors that may compromise adaptive repair, as well as their removal, is therefore paramount.

A common iatrogenic kidney insult in the hospital setting is medication with nephrotoxic potential—in one study 86% of non-critically ill children were exposed to this risk [3]. Frequently prescribed agents include antibiotics, antifungals, antivirals, non-steroidal anti-inflammatory agents, immunosuppressants, intravenous contrast media and angiotensin-converting-enzyme inhibitors. A systematic screening program in hospitalized children was successful in reducing medication-related AKI, the main benefit being more rapid recognition, which led care teams to reduce medication exposure earlier [56]. This initiative was followed by a sustained quality improvement program which demonstrated a reduction in medication-related AKI over a 3-year period [57]. These studies were not designed to assess the effect of this strategy on progression following AKI, however it is intuitive that early removal of nephrotoxic insults might facilitate adaptive repair and limit progression.

### Optimizing urine drainage

Relieving the urinary tract obstruction is a well-established management priority in AKI in both adults and children [6]. This is particularly important in children for whom congenital anomalies of the kidney and urinary tract (CAKUT) are a frequent cause of CKD. Children with CAKUT frequently experience episodes of AKI with acute-on-chronic impairment in kidney function. These episodes represent a high risk of progression. Renal tract dilatation should be evaluated using ultrasound [58], with optimization of urinary drainage in conjunction with a pediatric urologist and/or interventional radiologist if necessary. Delays in optimizing drainage can compromise adaptive repair.

### Controlling acid–base and electrolyte abnormalities

Acid–base homeostasis can be severely disrupted following AKI. Processes such as proximal tubular bicarbonate reabsorption, distal tubular proton excretion and medullary urea recycling are compromised. Severe acidosis can compromise the coordination of adaptive repair by disrupting protein charge, conformation and function. In the clinical setting, controlling acid–base disturbance is likely to facilitate adaptive repair.

Electrolyte dysregulation is a further feature of AKI that can disrupt cellular function and thus hamper adaptive repair. Careful attention to plasma electrolyte levels and correction of gross abnormalities may therefore ameliorate progression.

Renal replacement therapy (RRT) may be necessary to control electrolyte and acid–base disturbance in children with oligo-anuric AKI. The optimal timing and modality of RRT to reduce the risk of progression have not yet been determined. In critically ill children, earlier initiation of continuous RRT may improve survival [59]. A key principle for the RRT prescription is avoidance of rapid or excessive ultrafiltration as this may compromise intravascular volume and renal perfusion, thus hampering adaptive repair.

## Future therapies which may ameliorate progression following AKI

Whilst current clinical strategies to minimize progression focus on optimization of the microenvironment in order to facilitate adaptive repair, treatments to accelerate recovery are on the horizon. Potential targets for future therapies and their associated biochemical pathways are discussed in this section and summarized in Table 1.

**Table 1:** Pathways of progression following acute kidney injury and related mechanisms and therapeutic targets

Pathway	Mechanisms/model	Future therapies
Oxidative stress	Capillary rarefaction	Ischemic pre-conditioning Activation of hypoxia-inducible factor (HIF) Activation of nuclear factor erythroid 2-related factor 2 (Nrf2) Exogenous vascular endothelial growth factor
DNA damage response	Aberrant cell cycle arrest Endothelial to mesenchymal transition Epithelial to mesenchymal transition Pericyte to myofibroblast transition	p53 inhibitors
Epigenetic changes	Histone modifications DNA methylation Chromosomal conformational changes	Histone deacetylase inhibitors
Mitochondrial dysfunction	Suppression of mitochondrial biogenesis Toll-like receptor 4-dependent mitogen activated protein kinase Extracellular signal-regulated kinase signaling	Stimulators of mitochondrial biogenesis
Complement	Cell surface complement regulatory protein knockout	Complement pathway blockade

A key pathway with future therapeutic potential is that of hypoxia and oxidative stress, both factors which can mediate progression [23, 24]. A number of relevant therapeutic targets have been identified. HIFs are transcription factors that play a key role in mediating cellular responses to hypoxia. In animal models of AKI, pre-ischemic targeting of the HIF pathway is effective in ameliorating injury and progression in specific circumstances [26, 60]. Similarly, activation of Nrf2, a regulator of cellular resistance to oxidants, has shown promise in attenuating progression [61, 62]. Thirdly, exogenous VEGF, a stimulant of angiogenesis, can exert a protective effect on renal capillaries and ameliorate long-term damage following an acute insult [63]. The above targets have shown promise as potential future treatments that may minimize progression following hypoxic ischemic insults to the kidneys.

Mitochondrial dysfunction, which can result from AKI, presents an additional pathway relevant to progression. Suppression of mitochondrial biogenesis has been demonstrated in animal models of AKI [64] and is likely to promote progression to fibrosis through persistent cellular injury [65]. Therapeutic agents that support mitochondrial biogenesis and function may therefore be beneficial in ameliorating progression in the future.

Epigenetic changes can influence repair and progression following an acute kidney insult. Epigenetic alterations are activated after AKI and include histone modifications, DNA methylation and chromosomal conformational changes [16]. Novel therapeutic agents that target epigenetic alterations have shown promise in animal models of AKI; for example histone deacetylase inhibitors have been found to accelerate renal recovery and reduce fibrosis after AKI in zebrafish and mice [66].

The response of renal tubular epithelial cells to DNA damage is implicated in fibrosis of the kidney. Modifying the cell cycle of renal tubular epithelial cells via inhibition of tumor suppressor p53 and inhibiting downstream signaling from tubular cells in cell cycle arrest can prevent progression and fibrosis [67]. Further translational work is needed to determine if these strategies can ameliorate kidney fibrosis following AKI in the clinical setting.

Whilst the avenues discussed above hold promise for future accelerated adaptive repair in children at risk of AKI progression, they are all several steps away from being evaluated in the clinical setting. For now, the mainstays of clinical management to minimize progression remain optimization of renal perfusion, urinary drainage and the extracellular environment, and minimization of ongoing kidney insults.

## Summary

Acute kidney injury is common in children treated in hospital, and a significant proportion experience long-term sequelae. Following AKI, adaptive repair processes can result in the recovery of kidney function; conversely, maladaptive repair can lead to progression, defined as a durable change in kidney structure or function detected by biomarkers, imaging studies or histopathology.

Established risk factors for progression include severity of the kidney insult, the cumulative impact of multiple AKI episodes and pre-existing CKD. Further risk stratification using electronic health record data, biomarker panels and imaging modalities are being evaluated.

Clinical strategies aimed at minimizing progression include optimization of kidney perfusion, urinary drainage and electrolyte and acid–base balance, as well as prompt removal of ongoing kidney insults. Future therapies that may accelerate adaptive repair following AKI and ameliorate progression are on the horizon.

## Multiple-choice questions (answers are provided following the references)

1. Progression following AKI is defined as:
  - Increasing severity of acute kidney injury
  - Recovery of plasma creatinine to baseline
  - A durable change in kidney structure or function detected by biomarkers, imaging or histopathology
  - Extra-renal complications following AKI.
2. Following an acute kidney insult, which of the following are true?
  - Plasma creatinine may not change
  - injury initiates repair mechanisms
  - adaptive repair results in progression
  - progression can lead to chronic sequelae.
3. Risk factors for progression following AKI in children include:
  - Chronic kidney disease
  - Repeated episodes of AKI
  - Severity of the kidney insult
  - Interstitial fluid overload.
4. Clinical management priorities to reduce progression following AKI include:
  - Early angiotension-converting enzyme inhibition
  - Diuretic therapy
  - Optimizing kidney perfusion
  - All of the above.
5. Mechanistic pathways for future therapies include:
  - Oxidative stress
  - Cell cycle modification
  - Histone deacetylase inhibition
  - All of the above.
